# Phototaxis is a satiety-dependent behavioral sequence in *Hydra vulgaris*

**DOI:** 10.1242/jeb.247503

**Published:** 2024-09-25

**Authors:** Soonyoung Kim, Krishna N. Badhiwala, Guillaume Duret, Jacob T. Robinson

**Affiliations:** ^1^ Department of Electrical and Computer Engineering, Rice University, Houston, TX 77005, USA; ^2^ Department of Bioengineering, Rice University, Houston, TX 77005, USA; ^3^ Department of Neuroscience, Baylor College of Medicine, Houston, TX 77030, USA

**Keywords:** Animal behavior modeling, Extraocular, *Hydra vulgaris*, Internal states, Phototaxis, Satiety dependency

## Abstract

Understanding how internal states such as satiety are connected to animal behavior is a fundamental question in neuroscience. *Hydra vulgaris*, a freshwater cnidarian with only 12 neuronal cell types, serves as a tractable model system for studying state-dependent behaviors. We found that starved hydras consistently move towards light, while fed hydras do not. By modeling this behavior as a set of three sequences of head orientation, jump distance and jump rate, we demonstrate that the satiety state only affects the rate of the animal jumping to a new position, while the orientation and jump distance are unaffected. These findings yield insights into how internal states in a simple organism, *Hydra*, affect specific elements of a behavior, and offer general principles for studying the relationship between state-dependent behaviors and their underlying molecular mechanisms.

## Teaser

**Summary:** Phototaxis in *Hydra vulgaris* is satiety dependent, driven by specific changes in behavior.

## INTRODUCTION

Animal behavior is a dynamic process influenced by environmental cues and internal states (Hemingway et al., 2020; Luttbeg and Sih, 2010; Näslund and Johnsson, 2016). In neuroscience and ethology, uncovering the mechanisms by which complex behaviors are regulated can provide clues to the mechanics of decision making. Computational modeling in particular has been useful in identifying the components of behavioral patterns (Han et al., 2018; Naik et al., 2020; Wang et al., 2023) as well as investigating the underlying biomolecular and neural driving factors (Henson et al., 2007; Larson et al., 2018; van der Staay, 2006). However, the challenge in dissecting complex state-dependent behaviors to establish mechanistic models inevitably increases with the complexity of the anatomy, the connectome, and the breakdown of observable behavioral motifs.

*Hydra vulgaris*, a freshwater invertebrate, is a particularly tractable model organism for studying the mechanisms behind state-dependent behaviors thanks to its rudimentary anatomy, simple nerve net, and easily identifiable basal behaviors. The *Hydra* body has two layers of tissue, the ectoderm and endoderm, separated by an extracellular matrix with no apparent canonical organs (Technau and Steele, 2011). It exhibits sensorimotor reflexes to external stimuli such as temperature change (Bosch et al., 1988; Mast, 1903; Schroeder and Callaghan, 1981; Tzouanas et al., 2021; Yamamoto and Yuste, 2020), mechanical touch (Badhiwala et al., 2021; Mast, 1903; Rushforth et al., 1963; Wagner, 1905), chemicals (Badhiwala et al., 2018; Kepner and Hopkins, 1924; Lenhoff, 1961; Scappaticci et al., 2010) and light (Passano and McCullough, 1962; Plachetzki et al., 2012; Rushforth et al., 1963; Singer et al., 1963). Its radially symmetric nervous system consists of only a few thousand neurons in the form of a nerve net distributed throughout the entire body, with only 12 constituent neuronal subtypes (Primack et al., 2023; Siebert et al., 2019). Despite the diffuse neural architecture, there is evidence that neurons in the oral region (i.e. the head) are essential for information processing (Badhiwala et al., 2021). Hydras also possess a distinct and isolated behavioral repertoire, which includes longitudinal and radial contraction, elongation, nodding, feeding and somersaulting. These behaviors are sufficiently distinguishable that they can automatically be classified (Han et al., 2018). Moreover, functional neural circuits associated with specific behavioral motifs have been recently uncovered, providing opportunities to correlate behavior with neural activity (Dupre and Yuste, 2017). Given its position in the phylogenetic tree as a part of the sister group to bilaterians, studying the regulation of complex behaviors in *Hydra* can further develop our understanding of fundamental processes that have been conserved over millions of years.

One complex behavioral motif found in its natural state is somersaulting, which is its main mode of locomotion. During somersaulting, the *Hydra* anchors itself with its foot and sways around with its body column, head and tentacles until tumbling in the direction the head was pointing and moving its foot to a new location (Naik et al., 2020; Yamamoto and Yuste, 2023). An even more complex behavior is phototaxis, which is a goal-directed behavior: moving toward or away from a light source. This behavior was first described by Trembley in 1744 (see Lenhoff et al., 1986). It has since been documented in both the symbiotic green *Hydra* (e.g. *Hydra viridissima*) which bears photosynthetic algae, and in brown *Hydra* (e.g. *Hydra vulgaris*) (Bossert and Galliot, 2012; Pardy, 1976; Wilson, 1891). Feldman and Lenhoff (1960) suggested that phototaxis may be sensitive to food deprivation. This hypothesis was particularly intriguing because it implied not only that *Hydra* are capable of goal-directed behaviors but also that they change their behavior depending on their internal state. However, these reports remain observational, lacking a rigorous quantitative and systematic analysis to provide mechanistic explanations behind the potential state dependency.

Here, we show the first quantitative analysis and behavioral model of satiety-dependent phototaxis in wild-type AEP strain *Hydra vulgaris*. To perform this study, we developed a low-cost, high-throughput imaging setup to record the animals' behavior in the presence of a controlled light stimulus for an extended period of time. We discovered that hydras consistently move towards the light within 8 h when starved (i.e. 14 days post-feeding) but this phototaxis is attenuated when the animals are fed (i.e. 2 days post-feeding). These data suggest that satiety is a key internal state regulating phototaxis. Having established this relationship, we then asked which aspect of their phototactic behavior is specifically modulated by satiety. To answer this question, we first extracted three behavioral elements of phototaxis, namely head orientation, jump distance and jump rate. We found that satiety only changed the jump rate. Finally, we confirmed that the jump rate is solely responsible for the observed differences in phototaxis using a mathematical model that simulates this behavior using a simple algorithm with the aforementioned behavioral components.

## MATERIALS AND METHODS

### *Hydra vulgaris* strain and maintenance

All experiments were conducted on the *Hydra vulgaris* AEP strain. The animals were cultured using the protocol adapted from the laboratory of Robert Steele (University of California, Irvine, USA). Hydras were maintained at 18°C in an incubator on a 12 h:12 h light:dark cycle controlled by a Teensyduino, and were fed 3 times a week with freshly hatched *Artemia* nauplii (Brine Shrimp Direct). Hydras in the ‘starved’ group were starved for 14 days, and those in the ‘fed’ group were starved for 2 days prior to phototaxis experiments to provide ample time for complete digestion. Hydras were not reused unless explicitly stated otherwise.

### Microfluidic device fabrication and usage

All microfluidic chambers were fabricated using polydimethylsiloxane (PDMS; Sylgard 184). The master mold, adapted from previously designed lanes (Badhiwala et al., 2018), was 3D-printed (Formlabs Form3) to have 6 lanes per device. After the inlet and outlet ports were punched with a 1.25 mm diameter biopsy, the molded PDMS was O_2_ plasma-bonded to a 75 mm×50 mm glass slide (Corning 2947-75X50). After loading hydras into the device following a previously established protocol (Badhiwala et al., 2018; Tzouanas et al., 2021), the top surface of the device including ports was covered with optically clear adhesive well plate seals (Thermo Fisher Scientific AB1170) to minimize evaporation throughout the duration of the experiment. After each experiment, hydras were unloaded from the device, and the devices were flushed with deionized (DI) water and then soaked in 70% ethanol. After flushing the 70% ethanol out with DI water again, the devices were soaked in DI water and boiled at 150°C for ∼2 h, and completely dried on hot plates until future use.

### High-throughput long-term imaging setup

**Fig. 1.**
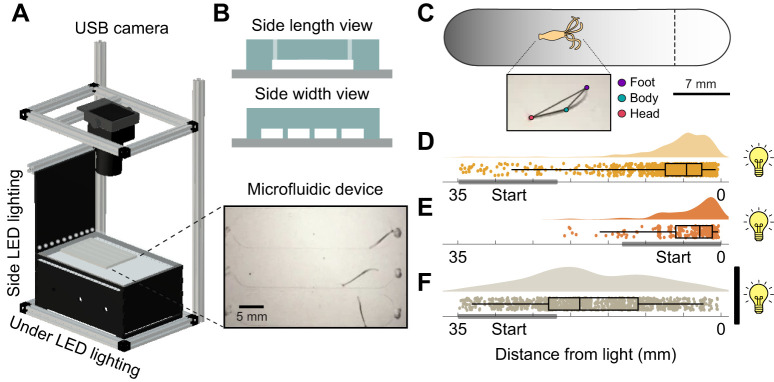
**Phototaxis is a robust and reproducible behavior in *Hydra vulgaris*.** (A) Schematic diagram of the high-throughput, long-term imaging setup. Teensyduino-controlled LEDs were used for both side and under illumination. USB cameras were connected to a computer for recordings. Microfluidic devices containing hydras in lanes (inset) were placed on the platform. (B) Microfluidic device overview. The top panel shows a schematic diagram of the typical side length view, which depicts how the microfluidic lanes are configured. The bottom panel shows the width view of how lanes are distributed within one device. (C) Schematic diagram of the phototaxis experiment. A representative optical gradient across the microfluidic lanes is shown. The dashed line indicates the threshold for phototaxis. The inset depicts the labeled body points derived from DeepLabCut. (D–F) Hourly foot location distribution of hydras when (D) placed on the darker side of the chamber and exposed to light (pooled from *N*=36), (E) placed on the brighter side of the chamber and exposed to light (pooled from *N*=6), and (F) placed on the darker side of the chamber with the light blocked (pooled from *N*=24). Box plots show median, first quartile and third quartile. The gray boxes in D–F show the range of the hydras' initial placement.

The lighting setup used in the experiment was built using a MakerBeam XL, a custom-made printed circuit board (PCB), LED strips (BTF-Lighting WS2812B), a commercial 8 megapixel USB camera (2.8–12 mm varifocal lens), and custom-cut acrylic casing (Fig. 1A,B). The MakerBeam profiles were used to build the skeleton, with a rectangular bottom and top structure connected by two vertical columns. One additional column and row was used for securing a side lighting panel. Acrylic was used to encase the under lighting panel and serve as a stage to place devices for imaging. An optical diffuser was attached to the stage and the side lighting panel for even diffusion. The LEDs were controlled with Teensyduino and TyCommander. The USB cameras were operated using iSpy software. Each setup was housed in a cabinet with doors in order to prevent external light leakage.

### Phototaxis assay

**Fig. 2.**
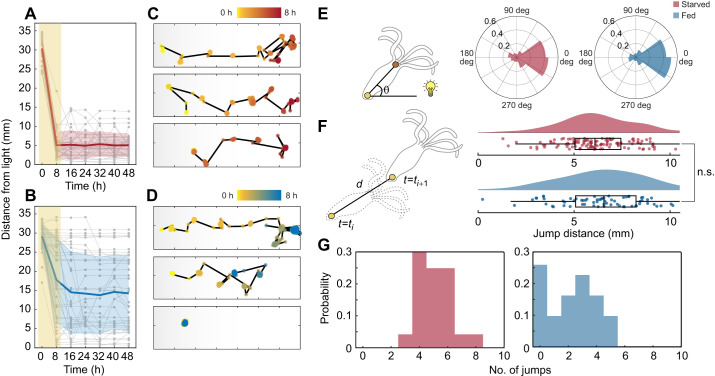
**Satiety attenuates the phototactic behavior in *Hydra* with reduced jump rate.** (A,B) Foot location of starved animals (*N*=24; A) and fed animals (*N*=31; B) over the course of 48 h, sampled at 0, 8, 16, 24, 32, 40 and 48 h time points. (C,D) Representative foot location traces from the first 8 h of the experiment, where starved (C) and fed (D) hydras were initially placed on the darker side of the chamber. (E) Left: calculation of head orientation (θ). Right: head orientation distribution of starved animals (pink) and fed animals (blue). Not significant, circular-analog Kruskal–Wallis test. (F) Left: definition of jump distance: *d*=*t_i_*_+1_–*t*_i_, *d* is the euclidian distance between the foot location of before (*t*_*i*_) and after (*t*_*i*+1_) a jump, where *t* is time and *i* is index for a specific time point. Right: jump distance distribution (*D*) of starved hydras (pink) and fed hydras (blue). n.s., not significant, Mann–Whitney *U*-test. (G) Probability distribution of the number of jumps of starved hydras (pink) and fed hydras (blue). *P*<0.001, Mann–Whitney *U*-test.

Prior to the start of the experiment, each microfluidic device with hydras inserted was placed on the imaging setup. For the experiments, we used white light from LEDs composed of red (R), green (G) and blue (B). We used one row of LEDs (10 LEDs) at maximum brightness (set as R:G:B 255:255:255) from the side lighting panel to provide uniform illumination that yielded an optical gradient across the length of the microfluidic lanes. At the edge of the chamber closer to the light, the power measured ∼4.15 mW. Lighting at reduced brightness (R:G:B 1:1:1) without any gradient was additionally used for experiments where the light from the side panel needed to be blocked, in order to facilitate video recording. Timelapse videos were recorded at 1 frame s^−1^ for 48 h. Foot coordinates (see ‘Behavioral analysis and generating synthetic phototaxis dataset’, below) were used to determine hydra location at a given time. The head orientation (θ) was calculated as the angle between the two vectors (Fig. 2E): (1) the vector connecting the foot to the hypostome, and (2) the vector connecting the foot to the light source. Jump distance (Fig. 2F) was calculated as the distance between the foot locations before and after a jump event. The number of jumps (Fig. 2G) is the number of jump events observed in each group.

### Behavioral analysis and generating the synthetic phototaxis dataset

DeepLabCut (Lauer et al., 2022) was used to quantify hydra body coordinates. Locations annotated were basal disk (foot), center of the body column (body) and the hypostome (head). Mislabeled frames were manually re-labeled. The coordinates were extracted every 20 frames from the videos, to reduce the number of repeated frames due to the hydras' slow movement. Custom MATLAB code was used for data analysis and modeling, including phototaxis simulations following the algorithm described in Results. For comparison between the experimental and synthetic dataset, only hydras that had the starting location between 23 and 35 mm away from the light source were selected.

### Statistical analysis

The circular-analog of the Kruskal–Wallis test for directional data (Circstat MATLAB package; Berens, 2009) was used to evaluate the statistical significance of head orientations (Fig. 2E). The Mann–Whitney *U*-test (Wilcoxon rank sum test) was used to evaluate statistical significance of jump distances and number of jumps (Fig. 2F,G).

## RESULTS

### Phototaxis is a robust and reproducible complex behavior in *Hydra vulgaris*

Although phototaxis had been reported anecdotally, a more definitive demonstration of phototaxis and its correlation with biological state required a more quantitative approach. One challenge is that *Hydra* move slowly (∼0.8 cm h^−1^). To account for their slow movements, we built a microfluidic setup that allows scalable and long-term imaging in a controlled environment (Fig. 1A,B; see Materials and Methods). We exposed the hydras placed in the microfluidic chamber to an optical gradient provided by a set of LEDs and an optical diffuser installed to one side of the chamber (Fig. 1A,C). We used DeepLabCut, a deep learning-based pose estimation algorithm (Lauer et al., 2022), to obtain and track the coordinates of the *Hydra* body parts, namely the foot, body column and head (Fig. 1C). Because *Hydra* mainly locomote by somersaulting, detaching the foot from the surface and attaching it to a new location, we used the foot coordinates to determine the location of the hydra (Naik et al., 2020; Yamamoto and Yuste, 2023)*.*

When initially placed on the darker side of the chamber, all hydras tested moved toward the light source (Fig. 1D). To verify that the phototaxis we observed was not an artifact of the animal's preference to move to the opposite end from where they were placed, we also tested placing animals initially on the brighter side of the chamber (i.e. closest to the light source). In these experiments, hydras stayed closer to the light source rather than moving away (Fig. 1E). Given that hydras respond to thermal stimulation, we also asked whether the behavior could be driven by a thermal gradient generated by the LEDs rather than an optical gradient. We repeated the experiment using an optical diffuser painted black, which allowed the thermal diffusion (1–2°C) to pass through while blocking the photon-emitting light. In this case, hydras roamed around the chamber without any apparent direction (Fig. 1F), suggesting that the phototaxis behavior we observed is not an artifact of thermal differences of the geometry of the chamber and is in fact light driven.

### Phototaxis in *Hydra* is satiety dependent

In the course of maintaining hydras, we observed that when we had not fed the animals recently, they seemed to be more attracted to light. This unexpected result led us to ask whether *Hydra* phototaxis is a satiety-dependent behavior. Considering that satiety level (i.e. hunger) is one of the major factors driving behavioral shifts across the animal kingdom (Bertsch et al., 2019; Gonzales et al., 2019; Landayan et al., 2018; Lin et al., 2019; Smith and Grueter, 2022), we sought to investigate whether this was also the case for phototaxis in *Hydra*. We divided a homogeneous *H. vulgaris* AEP population into two groups, namely ‘starved’ and ‘fed’. Animals in the starved group were not given any food for 14 days, while the hydras in the fed group were regularly fed until 2 days prior to phototaxis recordings. For each group, hydras were placed on the darker side of the chamber at the beginning of the experiment. We found that *Hydra* phototactic behavior was in fact satiety dependent. Only the starved hydras consistently locomoted towards the light within the first 8 h, and stayed within the vicinity afterwards (Fig. 2A; Movie 1). Fig. 2C shows representative traces of the foot locations over the course of the first 8 h, where the hydras move with strong directionality. In contrast, the phototactic behavior was drastically attenuated and variable in the fed animals (Fig. 2B; Movie 2). While some hydras displayed phototaxis, most either roamed with no apparent sense of direction with reduced movement or barely locomoted at all (Fig. 2D).

In order to identify behavioral components for the phototaxis, we focused our analysis on the first 8 h of the experiments, which we found to be sufficient for starved hydras to reach the light. We sought to identify key constituent behavioral parameters in phototaxis that were affected by the satiety level. *Hydra* mainly use somersaulting to move to a new location, tumbling in the direction the head was last pointing. It is also believed that photoreceptors or opsins are primarily located in the head and tentacles (Guertin and Kass-Simon, 2015; Macias-Muñoz et al., 2019), which are pertinent to light perception. Based on this prior knowledge, we extracted three parameters: head orientation, jump distance and jump rate.

First, we observed the head orientation with respect to the light source to determine with the distinct behavioral patterns stem from a difference in light perception between starved and fed populations. Head orientation (θ) was calculated using the coordinates obtained from DeepLabCut (Fig. 1C), as the angle between a vector connecting the hypostome and the foot, and a vector connecting the hypostome and the light source (Fig. 2E, left). We found that the differences in collective head orientation distribution for both starved and fed animals were indistinguishable (Fig. 2E, right), suggesting that the head orientation was not directly driving the behavioral difference. Next, we asked whether the shift in behavior arises from the difference in the jump distance. The jump distance (*d*) was calculated as the distance between the location of the foot before and after translocation. We observed no statistically significant difference between the jump distance distributions (*D*) of starved and fed animals (Fig. 2F). Rather, we found that the difference lies in the number of jumps (Fig. 2G), which translates to jump rate. Representative foot traces of starved and fed hydras (Fig. 2C,D) illustrate how starved animals generally display a higher number of jumps compared with fed hydras. Roughly half of the fed animals translocated 3 times or fewer per trial.

### Phototaxis in *Hydra* can be synthetically created using a reduced set of parameters

**Fig. 3.**
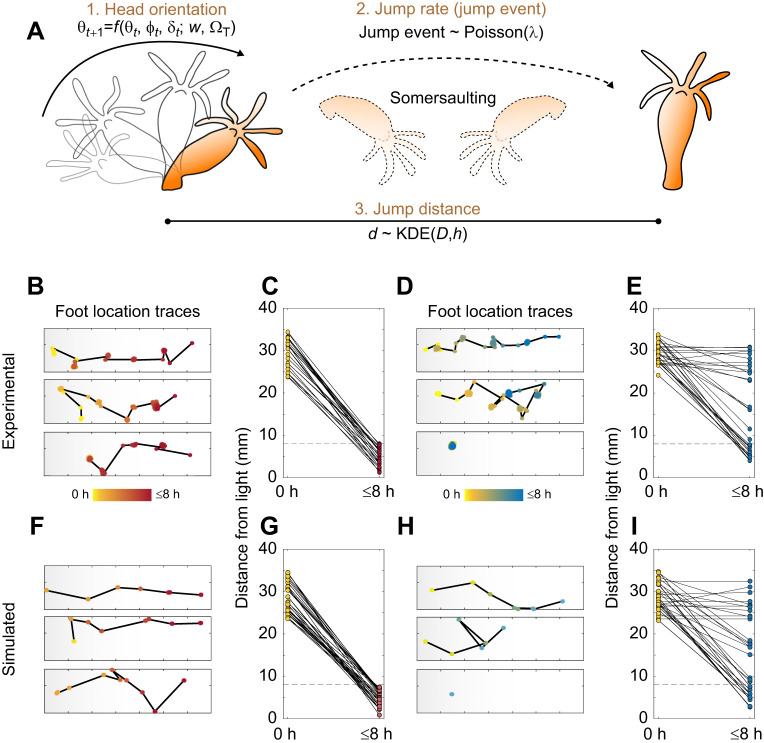
**Modeling phototaxis of *Hydra* shows the jump rate is a significant contributor to state-dependent behavioral change.** (A) Schematic diagram of the model using head orientation (1) with biased correlated random walk (BCRW), Poisson process for jump decision making (2) and jump distance distribution fitted from experimental data (3). θ, head orientation; *t*, time; φ and δ, noise; *w*, weight; Ω_T_, bias; λ, jump rate; *d*, jump distance; KDE, kernel density estimation; *D*, jump distance distribution; *h*, index for jump event. (B–E) Representative experimental foot location traces of (B) starved and (D) fed hydra; and experimental foot location from start (0 h) to termination (≤8 h) of (C) starved and (E) fed hydra (start location between 23 and 35 mm from light). (F–I) Simulated foot location traces of (F) starved and (H) fed hydra; and representative simulated foot location from start (0 h) to termination (≤8 h) of (G) starved animals (*N*=30) and (I) fed animals (*N*=30).

Having identified the jump rate as the most critical of the three behavioral parameters involved in phototaxis, we considered whether the jump rate alone was sufficient to explain satiety-dependent phototaxis. To answer this question, we used the three aforementioned parameters and a simple algorithm (Fig. S2) to generate synthetic phototaxis datasets. Each simulated ‘hydra’ was given a head orientation, a jump rate and a jump distance, where the next foot location was determined by this sequence for every iteration until it crossed the phototaxis threshold or reached the termination time of 8 h (Fig. 3A). Apart from the jump rate (λ), the parameters were the same between the synthetic starved and fed groups. Each synthetic hydra was given a starting point between 23 and 35 mm from the light source, as for the experimental data.

As the head orientation (θ) generally does not change abruptly (e.g. from 0 to 180 deg) with changes in orientation (Δθ) sharply centered on 0 deg (Fig. S1E), we adapted the concept of biased correlated random walk (BCRW) to model the head orientation. BCRW consists of two main terms: target that represents the bias (Ω_T_), and persistence that depicts correlation (Chudzinska et al., 2021; Lohmann et al., 2016; Whittington et al., 2004). The target in this case is the light source, and persistence is the probability of orienting the head in the direction of the previous head angle. Bias was given the weight *w* and persistence (1−*w*). Each term includes noise terms (ϕ and δ, respectively) as well, for which we used the truncated location scale distribution fitted from the experimental data, yielding:
(1)

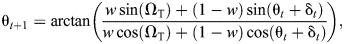
where *t* corresponds to a given iteration. After selecting the initial value for head orientation uniformly at random ranging from 0 to 360 deg, the progression followed the BCRW. The use of such a method resulted in similar head orientation trajectories between the experimental and simulated data for both starved and fed groups, indicating realistic modeling (Fig. S1A–D). The jump rate was calculated from the experimental data, which differed depending on the starved or fed population. Because of the varying behavior in the fed animals (Fig. 2B,D), with roughly half of the hydras executing a total of three jumps or fewer, we separately calculated jump rates for ‘active’ and ‘inactive’ fed hydras. Given the nature of the somersaulting behavior, where the time between jumps, or ‘events’, is random, we treated the phototactic movement as a Poisson process. Thus, the jump rate was calculated as λ, the Poisson variable, as follows:
(2)

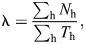
where *N*_h_ denotes the number of jumps for a given observed hydra, and *T*_h_ denotes the total time for the same given hydra. We also obtained the jump distance (*d*) from the distribution of the experimental data. As the jump distance distributions for starved (*D*_s_) and fed (*D*_f_) hydras were not statistically significantly different (Fig. 2F), the data points were pooled and fitted with a kernel density estimate using both data and bandwidth as inputs for sampling in simulations. When a jump event occurred, a jump distance was randomly sampled from the kernel-fitted pooled distribution *D*. In the case of no jump, the jump distance was set to zero. Using these set of parameters, the next foot location was determined using:
(3)

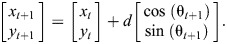
Using the aforementioned minimal set of three variables (θ; head orientation, *D*; jump distance, λ; jump rate) and a simple algorithm, we simulated *Hydra* phototactic behavior. Quantitatively, the foot location traces between the experimental starved hydras (Fig. 3B) and simulated starved hydras (Fig. 3F) were comparable, with apparent biased movement towards the light. The simulated hydras crossed the phototaxis threshold within the 8 h limit (Fig. 3G), mirroring the experimental data (Fig. 3C). Similarly, for fed hydras, the foot location traces were analogous between simulated and experimental fed hydras (Fig. 3D,H). The foot location distribution from the start (0 h) to the termination of simulation (≤8 h) confirmed similar patterns between the experimental (Fig. 3E) and simulated dataset (Fig. 3I), with two randomly assigned subpopulations of active and inactive fed hydras.

## DISCUSSION

The ability to shift behaviors depending on internal states found in many species is an important feature for animal survival. Here, we demonstrate that despite being phylogenetically distant from most model organisms in neuroscience and having a basic nervous system with a rudimentary anatomy, *H. vulgaris* is capable of performing complex goal-directed behaviors that are modulated by internal states. Prior to this work, phototaxis in brown *Hydra* such as *H. vulgaris* had only been described in anecdotal reports. Using a custom-designed imaging setup and microfluidic devices, we showed that *Hydra* indeed display robust phototaxis. More importantly, the phototactic behavior was strengthened in the starved population, whereas it was attenuated in the fed population, which indicates that there are state-dependent internal processes that modulate neural circuit activity and behavior. By breaking down the phototactic behavioral motif into individual elements, head orientation, jump distance and jump rate, we found that only the jump rate changed from starved to fed populations and confirmed that this change alone was sufficient to explain our data when we simulated *Hydra* phototaxis in our model. This result provides a specific behavioral change that derives from satiety and shows where future work can focus to understand the mechanistic and molecular underpinnings of this state change.

Despite this progress, we do not yet understand how *Hydra* sense where a light source is located. Prior reports have shown that *Hydra* can perceive and exhibit a sensorimotor reflex to light. Our results imply that *Hydra* can detect optical gradients without canonical visual organs and thus possibly only with photoreceptors (Feldman and Lenhoff, 1960; Musio et al., 2001; Taddei-Ferretti and Musio, 2000; Taddei-Ferretti et al., 1988, 2004). One possibility for future work is to test whether *Hydra* explore their environment by moving their photoreceptor-dense head and tentacles and storing the information over a short period of time before somersaulting toward the strongest light stimuli. This is particularly interesting because it suggests that *Hydra* possess memory-based decision-making capabilities. Future work should explore how *Hydra* compute optical gradients and decide to move towards the light source based on this computation, and why they stay at the brighter side of the chamber even after phototaxis is complete.

Another future direction is to investigate the biomolecular factors that drive satiety-dependent phototaxis in *Hydra* based on the demonstrated behavioral framework. The *Hydra* genome has been fully sequenced (Cazet et al., 2023; Chapman et al., 2010), and recent advances in molecular technology, especially single-cell RNA sequencing (scRNA-seq), provide a tremendous opportunity to probe the responsible genes and cell types that cause shifts in behavior (Chari et al., 2021; Chen et al., 2017). Changes in satiety are known to affect gene expression, which is correlated to changes in behavior. For example, hunger-driven changes in gene expression contribute to food preference and intake in *Drosophila* larvae (Ryuda et al., 2008). Feeding and starvation in *C. elegans* drives regulation of gene expression that affects fat storage and satiety behavior (Baugh and Hu, 2020; Hyun et al., 2016). In *Hydra*, changes in satiety are linked to changes in a number of genes including opsins (Giez et al., 2024). Single-cell sequencing in conjunction with an assay for transposase-accessible chromatin with high-throughput sequencing (ATAC-seq) can help identify the epigenetic factors (Cazet et al., 2023; Liu et al., 2020) and specific molecular signaling pathways. Based on these data, targeted genetic manipulations that modify phototactic behavior could help reveal the molecular mechanisms and pathways responsible for changes in phototactic activity. Together, these studies may help establish a comprehensive understanding of internal state-driven complex behaviors in a simple yet intricate organism.

With this future work, it may be possible to understand why *Hydra*, particularly when starved, are attracted to light. This phenomenon is observed in many organisms, including bacteria and other invertebrates (Gorostiza et al., 2016; Jékely, 2009). Light perception is deemed necessary for survival, such as finding food and shelter and avoiding predators (Fabian et al., 2024; Yela and Holyoak, 1997). The evidence that cnidocyte discharge in *Hydra* is mediated by optical input (Plachetzki et al., 2012), coupled with the fact that *Hydra* capture their prey using their cnidocytes, suggests that phototaxis in *Hydra* may be a result of food search. Similar to *Drosophila*, which have two distinct dopaminergic neural circuits that regulate foraging behaviors depending on the nutritional state (Landayan et al., 2018), there is a possibility that *Hydra* may also have a yet uncharacterized circuit that governs satiety-controlled behaviors.

Furthermore, comparison between *Hydra* phototaxis and stimulus-driven behaviors in other cnidarians can help uncover similarities and differences in sensory processing and locomotion, as well as how sensory information triggers motor behaviors. What is unique about *Hydra* is that they possess the ability to locomote freely even though they only have a polyp stage, while many other cnidarians in their polyp stages are sessile. *Hydra* locomotion via somersaulting is also notably different from pulsating jellyfish movement in their medusae stage or the peristaltic motion in *Nematostella vectensis*. One major aspect of the phototaxis behavior clearly is photoreception. Whether the organism possesses eyes like box jellyfish or not, like *Hydra*, there is evidence that the phototransduction pathway is involved in light perception (Fain et al., 2010; Koyanagi et al., 2008; Plachetzki et al., 2010; Van Wyk and Kleinlogel, 2023). The eyeless *Nematostella* larvae most likely have similar photosensing capabilities to *Hydra* as they respond to the light range from 315 to 650 nm although they do not exhibit canonical phototactic behavior (Lilly et al., 2024). Another eyeless organism, the larva of a coral *Pocillopora verrucosa*, has been reported to position itself vertically, influenced by light (Mulla et al., 2021). Because of the difference in the nature of the movement patterns, it would be difficult to compare the components of the behavior directly between organisms. However, it is interesting to note the sequence of the escape behavior seen in a sea anemone, *Stomphia didemon* (Dalby et al., 1988). The anemone seems to survey its surroundings with its tentacles, and when the predator touches the anemone, it detaches the pedal disk to translocate. Although this is different from phototaxis, using its oral region to detect sensory modalities and then moving in their direction bears similarity to the behavioral components of phototaxis in *Hydra*. Another layer that makes the phototaxis described in this paper particularly interesting is its dependency on satiety. Given that satiety is a major factor in behavioral changes across species, it is reasonable to speculate that satiety may change other cnidarians' behavior as well.

The aspect of this work that is most exciting to the authors is that *Hydra*, despite its relatively simple neuronal repertoire and architecture, is capable of goal-directed behaviors that are altered by internal states. The fact that this relatively sophisticated behavior is manifested in such a simple nervous system raises the prospect of developing a complete quantitative understanding of behavior that spans the molecular, cellular and organismal scales.

## Supplementary Material

10.1242/jexbio.247503_sup1 Supplementary information
