# Supplementary material for: Phototaxis is a satiety-dependent behavioral sequence in Hydra vulgaris
Source: J Exp Biol. 2024 Sep 25;227(18):jeb247503. doi: 10.1242/jeb.247503 (PMC11449437; doi:10.1242/jeb.247503)
Supplement: Supplementary information [file jexbio-227-247503-s1.pdf]

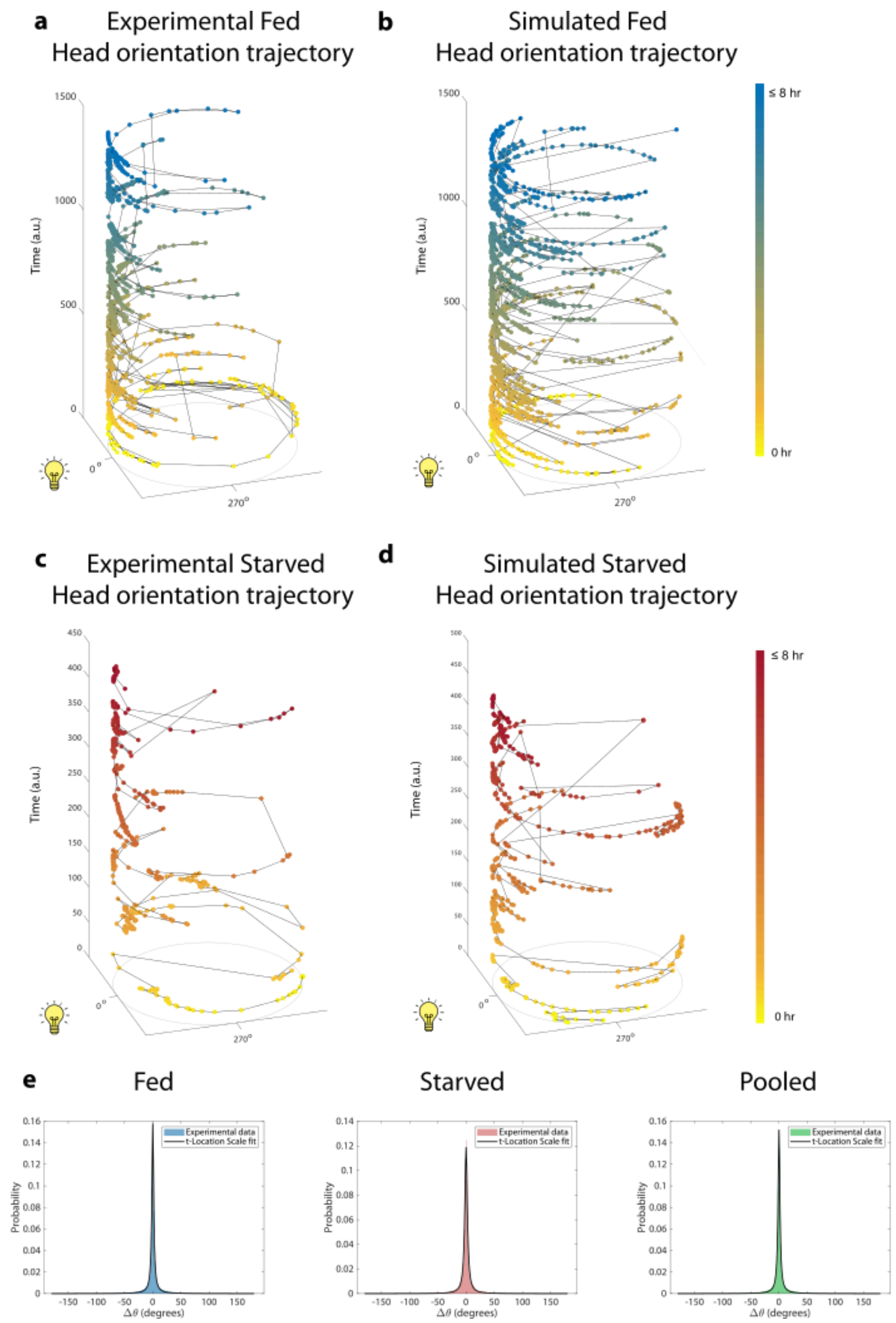

Fig. S1. Head orientation trajectories. (a) Head orientation trajectory of a representative experimental *fed Hydra*. (b) Head orientation trajectory of a representative synthetic *fed Hydra*. (c) Head orientation trajectory of a representative experimental *starved Hydra*. (d) Head orientation trajectory of a representative synthetic *starved Hydra*. (e) Differential head orientation for *fed* (left panel), *starved* (middle panel), and Pooled (right panel).

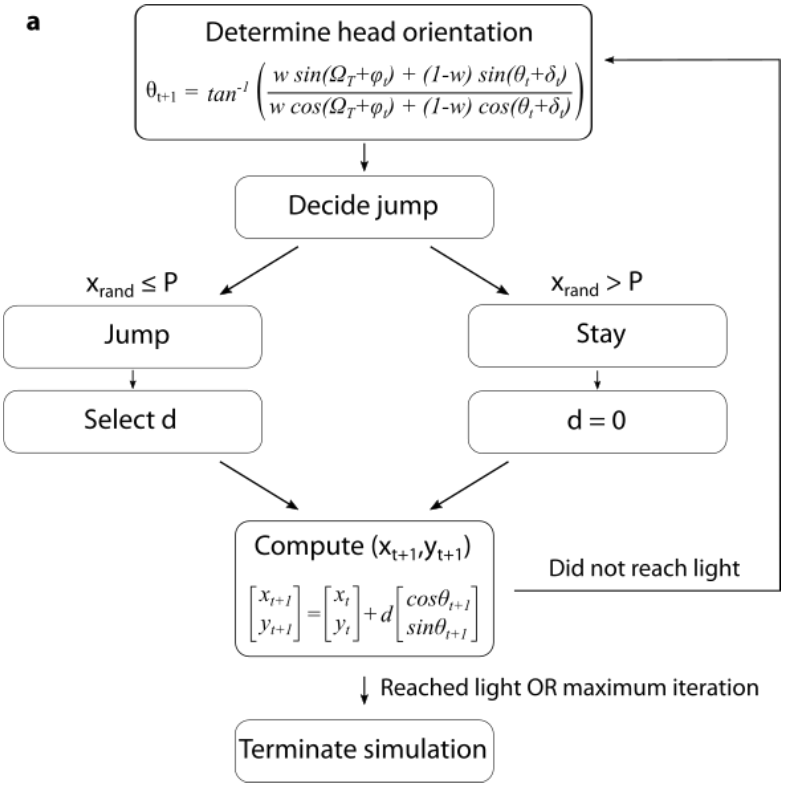

Fig. S2. Flowchart describing the modeling process

Journal of Experimental Biology • Supplementary information

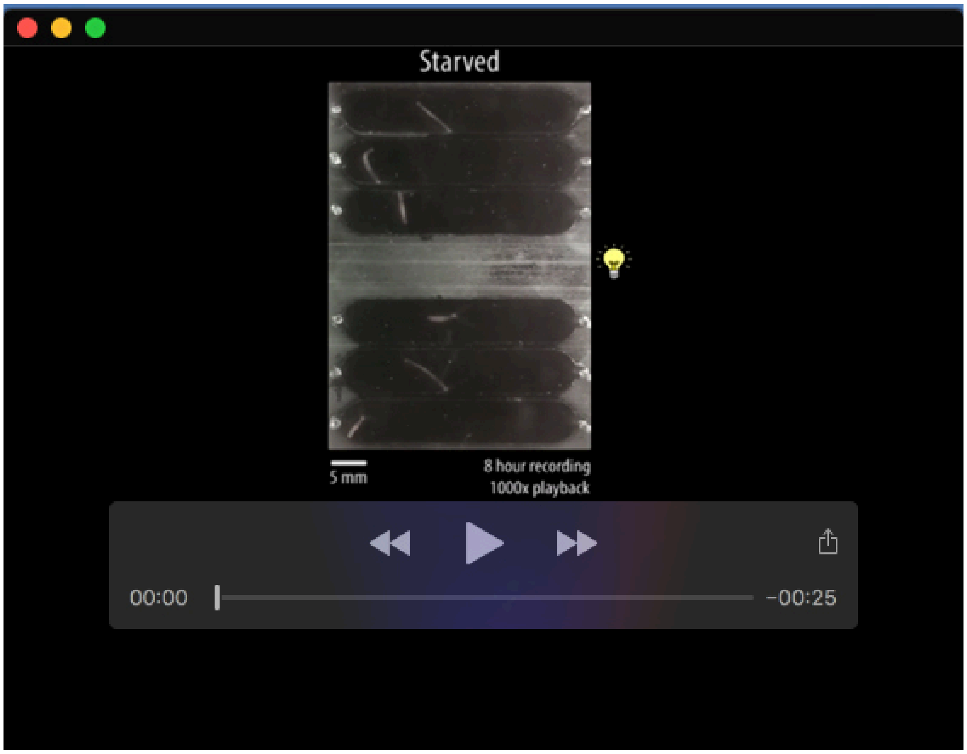

Movie 1. Video of starved *Hydra* recorded for 8 hours (1000x playback speed)

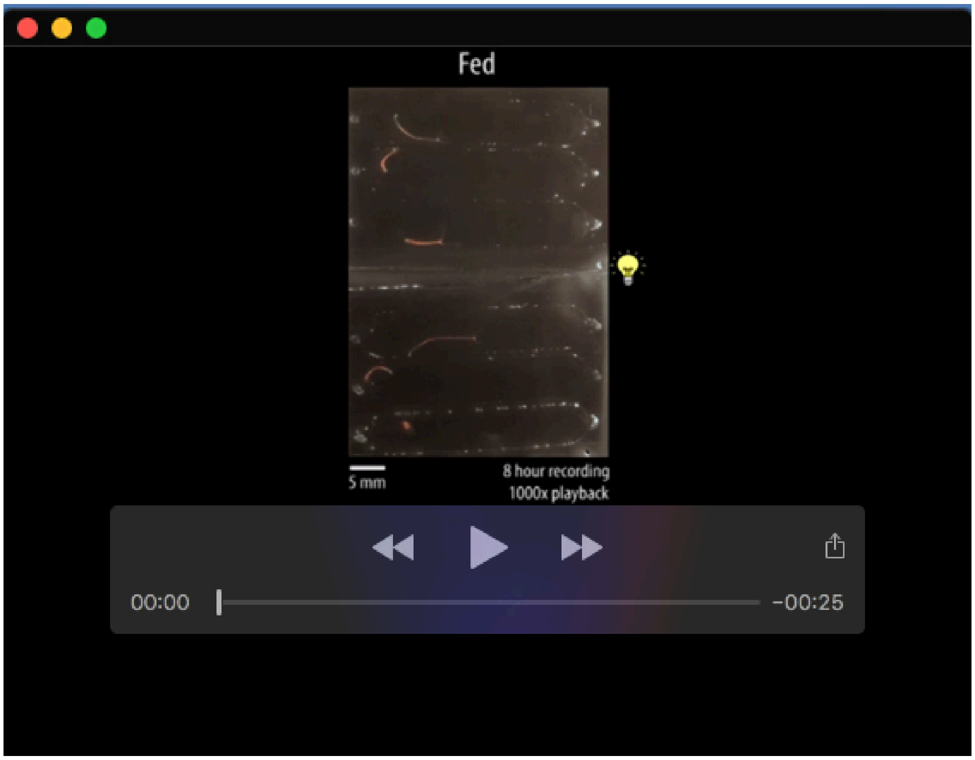

Movie 2. Video of fed *Hydra* recorded for 8 hours (1000x playback speed)

Journal of Experimental Biology • Supplementary information
